# Supplementary material for: Comparing Different Residential Neighborhood Definitions and the Association Between Density of Restaurants and Home Cooking Among Dutch Adults
Source: Nutrients. 2019 Aug 3;11(8):1796. doi: 10.3390/nu11081796 (PMC6722945; doi:10.3390/nu11081796)
Supplement: Supplementary file 1 [file nutrients-11-01796-s001.pdf]

Supplemental Digital Content

Table S1. Geo-FERN (Geographic Information System Food Environment ReportiNg) Checklist as proposed by Wilkins et al., (2016) [1]

Geo-FERN (Geographic Information System Food Environment ReportiNg) Checklist		
INSTRUCTIONS		
For each reporting item, insert a tick or cross in the shaded box to indicate whether the item has been reported, or insert 'N/A' if not applicable. Shading indicates whether items are essential or desirable. Reporting items can be included in supplementary materials if word limits are tight and if allowed by the publisher.		
1. FOOD OUTLET DATA	Essential	Desirable
Name of the data creator (e.g. 'Yellow Pages', 'Dunn & Bradstreet' etc.).	✓	
Collection and/or publication year of the data (include both if known).	✓	
Title of the dataset.	N/A	
Digital identifier of the dataset (e.g. a web address or DOI).		N/A
Publisher of the dataset.		✓
Scope of the dataset (i.e. the geographic coverage of the dataset e.g. 'national' or 'regional' and the range of businesses included in the dataset, including any notable exclusions).		✓
Identification of the data fields used in analyses.		✓
Original purpose of the data (e.g. food hygiene regulation enforcement or commercial business data).		✓
Methods used by the data creator to collect the data/compile the dataset (e.g. audits conducted by data creator).		✓
Prevalence of missing data (e.g. number of entries with incomplete address information).		
Methods for handling missing data (e.g. case-wise deletion, or use of secondary sources to impute missing data).		✓
Information on the accuracy of the data e.g. via reference to one or more validation studies or acknowledgement that data accuracy is unknown.		✓
2. EXTRACTING FOOD OUTLETS	Essential	Desirable
Description of methods used to extract food outlets of interest from dataset (e.g. search for specific proprietary classifications or store names).	✓	
If outlets were extracted using search terms (e.g. proprietary classifications or store names):<br>• An exhaustive list of search terms (where proprietary classifications are used, it should be made explicitly clear that the classifications listed are those of the data provider).	N/A	
If outlets were extracted based on proprietary classifications:<br>• A copy of the proprietary classification scheme, optionally including exemplary outlets falling within each classification; OR,<br>• A discussion of any notable categories excluded from analyses (e.g. pubs, pharmacies, mobile food vendors etc.).		✓
3. DEFINING FOOD OUTLET CONSTRUCTS	Essential	Desirable
Construct name(s) (e.g. 'supermarkets', 'healthy outlets', 'convenience stores' etc.).	✓	
Description of the methods used to group outlets into constructs, including at least one of:	✓	

• An *exhaustive* list of any list-based criteria used to define each construct. This could include e.g. proprietary classifications making up each construct, or a list of store names making up each construct. Where proprietary classifications are used, it should be made explicitly clear that the classifications listed are those of the data provider.<br>• Any objective criteria e.g. floor space, number of tills etc. used to define constructs.<br>• Citation of any previously published categorisation schemes that have been applied to the data and description of the methods used to apply the scheme.<br>• Description of any other methods used (note methods based on subjective criteria are discouraged).		
Examples of outlets falling within each construct such that the scope of each construct can be more readily interpreted. For example, if the construct 'fast food outlet' includes 'traditional' burger and fried chicken outlets, and also coffee shops and sandwich shops then well-known chains falling [illegible]		
Identification of any additional data sources used to group outlets into constructs e.g. use of Google Street View, business directories etc.		N/A
Description of how any additional data sources were linked to the food outlet data (e.g. by matching store names and/or addresses).		N/A
Where proprietary classifications are used to define constructs, a copy of the entire proprietary classification scheme.		
4. GEOCODING METHODS	Essential	Desirable
Acknowledgement of whether any data has been geocoded.	✓	
The address model used (e.g. areal unit, street segment, land parcel, address point).	✓	
The match rate achieved.	N/A	
The environmental context, including details on how this was defined e.g. the study area was urban/rural, defined based on population density.	✓	
Geocoding software used, including the version number.	✓	
The source of geocoding reference data (e.g. street line segment data), including publication date.		
5. ACCESS METRICS	Essential	Desirable
Definition of the conceptual environment being measured e.g. home, school, work etc.	✓	
Intensity Metrics		
If areal zoning system used:<br>• The type of areal zoning system (e.g. government districts, census tracts etc.)<br>• The source of boundary data, including the publication date or other version identifier.	✓	
If buffer zoning system used:<br>• The buffer size.<br>• The type of distance measure (e.g. Euclidian or network).	✓	
The units of the intensity metric(s) (e.g. count per unit area, as measured in meters) or formula indicating how they were calculated.	✓	
If network data was used (i.e. to calculate network distances):<br>• The source and publication date of network data.<br>• The types of road/path included.	N/A	

Rationale for the choice of zone type (e.g. areal vs buffer) and/or size as applicable.		✓
Proximity Metrics		
The type of distance measure (Euclidian vs network).	N/A	
If network data was used (i.e. to calculate network distances):<br>• The source and publication date of network data.<br>• The types of road/path included.	N/A	
Gravity Metrics		
The zone radius.	N/A	
The decay coefficient.	N/A	
6. UNKNOWN DETAILS	Essential	Desirable
Any items noted as essential, but that are unknown should be highlighted as a limitation.	N/A	

[1] Wilkins, E.L.; Morris, M.A.; Radley, D.; Griffiths, C. Using geographic information systems to measure retail food environments: Discussion of methodological considerations and a proposed reporting checklist (geo-fern). Health Place 2017, 44, 110-117, https://doi.org/10.1016/j.healthplace.2017.01.008.

Table S2. Sensitivity analyses performed only with individuals who drew self-defined neighbourhoods that were within the range of area sizes for administrative neighbourhood boundaries (0.25 to 4.14 km²) (n=720).

		Frequency of home cooking (6 -7 days per week)		
**Density of restaurants within self-defined neighbourhoods area of 0.25 to 4.14 km^2**		Model 1	Model 2	Model 3
		IRR (95% CI)	IRR (95% CI)	IRR (95% CI)
Self-defined neighbourhoods	T1 (lowest)	1	1	1
	T2	0.98 (0.88 – 1.07)	1.02 (0.92 – 1.12)	1.02 (0.92 – 1.13)
	T3 (highest)	0.93 (0.83 – 1.04)	1.08 (0.93 – 1.26)	1.07 (0.90 – 1.26)

T1, T2 and T3 are tertiles of densities, where individuals in T1 have the lowest density of restaurants and individuals in T3 the highest density; All models were performed individually; Model 1: adjusted for age, sex, education, income, household composition, employment status, spare time spent in the neighbourhood, years of residency in the neighbourhood, presence of restaurants was a reason for choosing the neighbourhood. Model 2: additionally adjusted for the density of grocery stores. Model 3: additionally adjusted for the density of all other non-restaurants food retailers.

Table S3. Descriptive characteristics of the SPOTLIGHT participants according to exclusion criteria.

Characteristics	Full sample n=6,037		Dutch participants N=1,609		Participants with self-defined neighbourhood n=1,272	
	n		n		n	
**Weekly frequency of cooking at home (%)**	5,456		1,431		1,164	
6 -7 days per week		65.0		73.2		74.1
**Age - mean (SD)**	5,975	51.8 (16.4)	1,598	54.9 (15.9)	1,266	53.9 (15.8)
**Sex (%)**	5,977		1,595		1,263	
Female		56.0		54.0		52.7
**Educational attainment (%)**	5,410		1,453		1,181	
Lower		11.6		16.8		14.3
Medium		34.8		26.8		25.7
Higher		53.6		56.4		60.0
**Income**	3,399		1,369		1,118	
Lower		26.2		30.0		28.2
Medium		18.5		21.8		21.3
Higher		55.3		48.2		50.5
**Household composition (%)**	5,330		1,439		1,174	
1 person		22.6		26.1		26.3
2 persons		39.2		43.3		41.7
3 or more persons		38.2		30.6		32.0
**Employed or in education (%)**	6,014		1,604		1,269	
Yes		58.5		56.9		60.7
**Spend most of spare time in the neighbourhood**	5,852		1,556		1,251	
Yes		72.0		76.9		76.0
**Presence of restaurant is a reason for living in the neighbourhood**	4,206		1,225		1,007	
Yes		17.6		14.7		14.5
**Years of residency in the neighbourhood**	5,800		1,564		1,259	
10 or more years		63.7		64.3		63.8

Table S4. Incidence Rate Ratio (IRR) and 95% confidence intervals (95%CI) as derived from Poisson regression analyses indicating the associations between *the study covariates* with weekly frequency of cooking at home among adults in the Netherlands. The SPOTLIGHT Project (n=1,245).

	**Frequency of home cooking (6 -7 days per week)**			
Covariates	800-metre buffer	1600-metre buffer	Administrative neighbourhood	Self-defined neighbourhood
	IRR (95% CI)	IRR (95% CI)	IRR (95% CI)	IRR (95% CI)
Density of all other food retailers	0.99 (0.98 – 1.00)	0.99 (0.98 – 1.00)	1.00 (0.99 – 1.00)	1.00 (0.99 – 1.00)
Age	1.00 (1.00 – 1.01)	1.00 (1.00 – 1.01)	1.00 (1.00 – 1.01)	1.00 (1.00 – 1.01)
Sex (female)	1.15 (1.07 – 1.23)	1.15 (1.07 – 1.23)	1.15 (1.07 – 1.24)	1.15 (1.07 – 1.24)
Educational attainment	1.10 (1.04 – 1.17)	1.10 (1.04 – 1.17)	1.10 (1.04 – 1.17)	1.10 (1.04 – 1.17)
Income	1.00 (0.95 – 1.06)	1.00 (0.95 – 1.06)	1.00 (0.95 – 1.06)	1.02 (0.95 – 1.07)
Household Composition	1.21 (1.14 – 1.29)	1.22 (1.14 – 1.29)	1.22 (1.15 – 1.30)	1.23 (1.15 – 1.30)
Employment or in education	0.89 (0.81 – 0.96)	0.89 (0.82 – 0.97)	0.89 (0.81 – 0.97)	0.89 (0.81 – 0.97)
Most of spare time in the neighbourhood	1.00 (0.93 – 1.09)	1.00 (0.93 – 1.09)	1.00 (0.93 – 1.09)	1.00 (0.93 – 1.09)
Restaurant was a reason for choosing the neighbourhood	0.82 (0.72 – 0.95)	0.82 (0.71 – 0.95)	0.82 (0.71 – 0.95)	0.81 (0.71 – 0.94)
Years of residency in the neighbourhood	0.96 (0.89 – 1.04)	0.96 (0.89 – 1.04)	0.96 (0.89 – 1.04)	0.96 (0.89 – 1.04)

Model 3: adjusted for age, sex, education, income, household composition, employment status, spare time spent in the neighbourhood, years of residency in the neighbourhood, presence of restaurants was a reason for choosing the neighbourhood and additionally adjusted for the density of all other food retailers.

Table S5. Incidence Rate Ratio (IRR) and 95% confidence intervals (95%CI) as derived from Poisson regression analyses indicating the associations *of the joint* effects of exposures and the potential effect modifiers *and their* product term with weekly frequency of cooking at home among adults in the Netherlands. The SPOTLIGHT Project (n=1,245).

	**Frequency of home cooking (6 -7 days per week)**			
Multiplicative interactions	800-metre buffer	1600-metre buffer	Administrative neighbourhood	Self-defined neighbourhood
	IRR (95% CI)	IRR (95% CI)	IRR (95% CI)	IRR (95% CI)
**Age in two categories**				
65 years old or more	1.17 (1.03 – 1.33)	1.16 (1.02 – 1.31)	1.26 (1.10 – 1.45)	1.18 (1.03 – 1.34)
**Density of restaurant**				
Medium density	1.05 (0.96 – 1.16)	0.95 (0.86 – 1.04)	1.08 (0.98 – 1.18)	1.00 (0.91 – 1.09)
High density	1.04 (0.91 – 1.19)	0.97 (0.87 – 1.08)	1.05 (0.93 – 1.20)	0.94 (0.84 – 1.06)
**Age x Density of restaurant**				
65 years old or more x Medium density	0.98 (0.83 – 1.14)	1.00 (0.85 – 1.19)	0.86 (0.73 – 1.00)	0.95 (0.80 – 1.12)
65 years old or more x High density	1.04 (0.84 – 1.30)	1.03 (0.85 – 1.25)	0.96 (0.75 – 1.23)	1.07 (0.88 – 1.30)
**Education in three categories**				
Medium education	0.89 (0.73 – 1.09)	0.93 (0.77 – 1.12)	0.95 (0.80 – 1.12)	0.97 (0.81 – 1.16)
Higher education	1.05 (0.88 – 1.24)	1.08 (0.92 – 1.28)	1.08 (0.93 – 1.26)	1.10 (0.93 – 1.30)
**Density of restaurant**				
Medium density	0.89 (0.71 – 1.12)	0.89 (0.72 – 1.11)	0.96 (0.75 – 1.23)	0.99 (0.79 – 1.24)
High density	0.98 (0.68 – 1.19)	0.81 (0.60 – 1.10)	0.85 (0.62 – 1.16)	0.81 (0.61 – 1.08)
**Education x Density of restaurant**				
Medium education x Medium density	1.24 (0.94 – 1.64)	1.10 (0.83 – 1.45)	1.06 (0.78 – 1.45)	0.97 (0.73 – 1.28)
Medium education x High density	1.08 (0.77 – 1.52)	1.11 (0.76 – 1.58)	1.14 (0.78 – 1.66)	1.13 (0.80 – 1.60)
Higher education x Medium density	1.16 (0.92 – 1.47)	1.05 (0.83 – 1.32)	1.07 (0.82 – 1.39)	1.01 (0.79 – 1.28)
Higher education x High density	1.21 (0.91 – 1.60)	1.25 (0.92 – 1.69)	1.26 (0.91 – 1.74)	1.24 (0.92 – 1.67)

**Years of residency in the neighbourhood in two categories**				
10 years or more	1.01 (0.90 – 1.14)	1.02 (0.92 – 1.13)	1.00 (0.89 – 1.13)	0.97 (0.87 – 1.09)
**Density of restaurant**				
Medium density	1.05 (0.92 – 1.20)	0.97 (0.84 – 1.11)	1.02 (0.90 – 1.16)	0.98 (0.86 – 1.11)
High density	1.16 (0.98 – 1.37)	1.08 (0.94 – 1.23)	1.11 (0.95 – 1.30)	0.99 (0.85 – 1.14)
**Residency years x Density of restaurant**				
10 years or more x Medium density	0.99 (0.85 – 1.16)	0.97 (0.82 – 1.15)	1.00 (0.85 – 1.17)	1.00 (0.86 – 1.17)
10 years or more x High density	0.85 (0.71 – 1.02)	0.86 (0.73 – 1.01)	0.87 (0.72 – 1.06)	0.96 (0.81 – 1.13)
**Income in three categories**				
Medium income	0.89 (0.72 – 1.09)	1.02 (0.84 – 1.23)	0.99 (0.83 – 1.17)	1.03 (0.85 – 1.25)
Higher income	0.88 (0.75 – 1.05)	1.00 (0.85 – 1.17)	0.95 (0.81 – 1.11)	1.05 (0.88 – 1.24)
**Density of restaurant**				
Medium density	0.93 (0.77 – 1.11)	1.04 (0.87 – 1.24)	1.03 (0.86 – 1.24)	1.06 (0.89 – 1.28)
High density	0.87 (0.68 – 1.11)	0.88 (0.70 – 1.10)	0.92 (0.74 – 1.14)	0.95 (0.77 – 1.17)
**Income x Density of restaurant**				
Medium income x Medium density	1.13 (0.87 – 1.46)	0.85 (0.65 – 1.12)	0.94 (0.72 – 1.23)	0.96 (0.73 – 1.26)
Medium income x High density	1.16 (0.85 – 1.57)	1.02 (0.77 – 1.36)	1.00 (0.77 – 1.34)	0.92 (0.69 – 1.23)
Higher income x Medium density	1.15 (0.94 – 1.40)	0.87 (0.71 – 1.07)	1.00 (0.82 – 1.21)	0.89 (0.72 – 1.10)
Higher income x High density	1.28 (1.00 – 1.62)	1.18 (0.93 – 1.49)	1.24 (0.99 – 1.56)	1.06 (0.85 – 1.33)

Model 3: adjusted for age, sex, education, income, household composition, employment status, spare time spent in the neighbourhood, years of residency in the neighbourhood, presence of restaurants was a reason for choosing the neighbourhood and additionally adjusted for the density of all other food retailers.

Table S6. Sensitivity analysis using 5 categories for the exposure measures showing Incidence Rate Ratio (IRR) and 95% confidence intervals (95%CI) as derived from Poisson regression analyses indicating the associations between density of restaurants, according to four different definitions of neighbourhoods, with weekly frequency of cooking at home among adults in the Netherlands. The SPOTLIGHT Project (n=1,245).

		**Frequency of home cooking (6 -7 days per week)**		
**Density of restaurants**		Model 1	Model 2	Model 3
		IRR (95% CI)	IRR (95% CI)	IRR (95% CI)
800m buffers	Q1 (lowest)	1	1	1
	Q2	0.99 (0.90 – 1.09)	0.98 (0.90 – 1.08)	0.99 (0.90 – 1.09)
	Q3	0.97 (0.88 – 1.07)	1.00 (0.90 – 1.11)	0.99 (0.90 – 1.10)
	Q4	1.02 (0.92 – 1.12)	1.06 (0.95 – 1.18)	1.05 (0.94 – 1.17)
	Q5 (highest)	0.91 (0.80 – 1.03)	1.06 (0.86 – 1.30)	1.01 (0.83 – 1.23)
1600m buffers	Q1 (lowest)	1	1	1
	Q2	0.95 (0.87 – 1.05)	0.96 (0.87 – 1.05)	0.95 (0.87 –1.05)
	Q3	0.97 (0.88 – 1.07)	0.98 (0.88 – 1.09)	0.97 (0.88 – 1.07)
	Q4	0.96 (0.87 – 1.05)	0.97 (0.87 – 1.07)	0.96 (0.87 – 1.06)
	Q5 (highest)	0.88 (0.77 – 1.00)	0.92 (0.74 – 1.14)	0.89 (0.71 – 1.10)
Administrative neighbourhoods	Q1 (lowest)	1	1	1
	Q2	1.00 (0.90 – 1.11)	0.99 (0.89 – 1.10)	1.00 (0.90 – 1.11)
	Q3	1.00 (0.92 – 1.09)	1.03 (0.94 – 1.12)	1.02 (0.93 – 1.11)
	Q4	1.04 (0.92 – 1.17)	1.02 (0.91 – 1.15)	1.05 (0.93 – 1.20)
	Q5 (highest)	0.93 (0.82 – 1.05)	1.02 (0.86 – 1.22)	0.99 (0.81 – 1.21)
Self-defined neighbourhoods	Q1 (lowest)	1	1	1
	Q2	0.93 (0.79 – 1.11)	0.93 (0.79 – 1.11)	0.93 (0.79 – 1.11)
	Q3	1.00 (0.92 – 1.09)	0.99 (0.91 –1.08)	0.99 (0.91 – 1.08)
	Q4	1.02 (0.94 – 1.11)	1.00 (0.92 – 1.11)	1.00 (0.90 – 1.10)
	Q5 (highest)	0.91 (0.81 – 1.03)	0.89 (0.77 – 1.03)	0.87 (0.75 – 1.01)

Q1, Q2, Q3, Q4 and Q5 are quintiles of densities, where individuals in Q1 have the lowest density of restaurants and individuals in Q5 the highest density; Model 1: adjusted for age, sex, education, income, household composition, employment status, spare time spent in the neighbourhood, years of residency in the neighbourhood, presence of restaurants was a reason for choosing the neighbourhood. Model 2: additionally adjusted for the density of grocery stores. Model 3: additionally adjusted for the density of all other food retailers.
